# Electrocortical effects of MDMA are potentiated by acoustic stimulation in rats

**DOI:** 10.1186/1471-2202-7-13

**Published:** 2006-02-16

**Authors:** Michelangelo Iannone, Stefania Bulotta, Donatella Paolino, Maria Cristina Zito, Santo Gratteri, Francesco S Costanzo, Domenicantonio Rotiroti

**Affiliations:** 1 CNR – Institute of Neurological Science, Section of Pharmacology, Catanzaro, 88021, Roccelletta di Borgia, Catanzaro, Italy; 2 Faculty of Pharmacy, University "Magna Græcia" of Catanzaro, Catanzaro, 88021, Roccelletta di Borgia (CZ) Catanzaro, Italy; 3 Faculty of Medicine and Surgery, University "Magna Græcia" of Catanzaro, Viale Europa, Località Germaneto, Catanzaro, Italy

## Abstract

**Background:**

3,4-Methylenedioxymethamphetamine (MDMA; ecstasy) is known for its toxicological, psychopathological and abuse potential. Some environmental conditions, e.g. acoustic stimulation typical of the "rave scene" can influence the toxicity of this drug.

**Results:**

We investigated the effects of low doses of MDMA in vivo using Wistar rats in the absence of acoustic stimulation (white noise; 95 Db) demonstrating that ecstasy is able to induce a significant activation (reduction of Electrocortical total power) of the telencephalic cortex that spontaneously reverts in the absence of sensorial stimuli, whereas it persists for several days if, in addition to MDMA, the animals are exposed to acoustic stimulation.

**Conclusion:**

Our data demonstrate that low doses of MDMA are able to reduce electrocortical total power, and that this effect is potentiated by sensorial stimuli commonly present in certain environments, such as rave parties.

## Background

The use of illicit drugs such as 3,4-Methylenedioxymethamphetamine (MDMA; ecstasy) has increased among young people in Europe and North America [1,2] over the past years.

Concern has been expressed about the increasing popularity of this stimulant drug and its association with certain youth subcultures, in particular the dance music scene [3].

The widespread use of ecstasy is due to its ability to produce feelings of euphoria and energy and a desire to socialize. In addition to these positive effects, MDMA is relatively inexpensive to produce and purchase and has the reputation of being safer than other recreational drugs.

Yet there is mounting evidence that ecstasy does not deserve this rosy reputation. In fact, evidence has been accumulated, both in human and animal studies, that shows the possible risks engendered by the consumption of MDMA [4]. In many reviews these risks are extensively discussed in terms of toxicity, psychopathology and abuse potential associated with acute and chronic use [5].

It is also clear that some environmental conditions can influence the toxicity of this drug in humans. For example, one of the consequences of the use of ecstasy at "raves" is the increase in body temperature that is due to a direct action of the drug on the thermoregulatory system, to the intense muscular activity and to elevated environmental temperatures. In addition, evidence from research done with an assortement of animal species from rodents to non-human primates, has shown that ecstasy is neurotoxic [5]. In fact, it has been shown that ecstasy is able to cause serotonergic [6,7] and dopaminergic [8] neuronal toxicity in every animal species tested and short-term changes in the noradrenergic system [5].

It has also been repeatedly demonstrated that electroencephalography may be a cheap and effective tool for examining neurotoxic effects of MDMA in humans where ecstasy use is positively correlated with absolute power changes in some frequency bands [4,9].

One of the questions which need addressing by research is how other factors typical of the "rave scene", such as sensorial auditory (techno music) stimuli, can affect higher neural functions and in particular electrocortical activity [10].

Based on these evidences, we investigated whether sound stimulation affects electrocorticographic changes of total spectrum power induced by simultaneous administration of low doses of MDMA in rats.

## Results

### Short term evaluation

**Figure 1:**
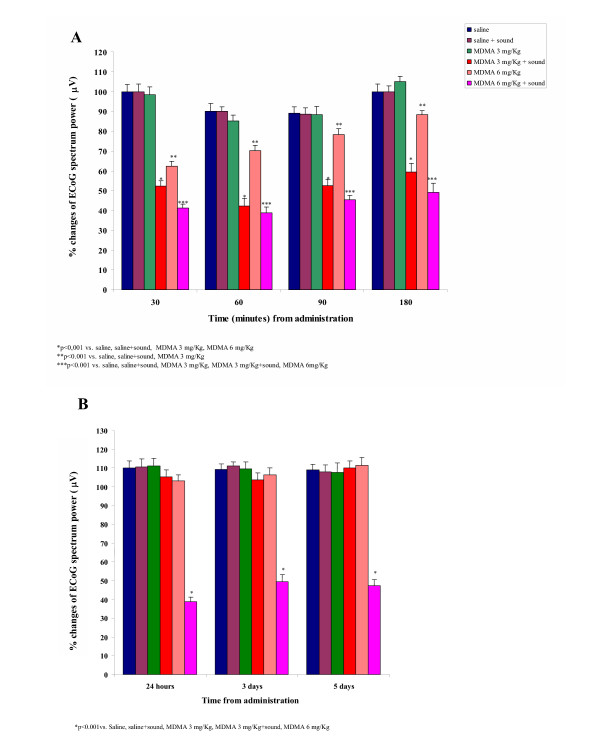
**The effects of MDMA and acoustic stimulation on ECoG power spectrum**. Effects of MDMA administration in the presence or absence of sound stimulation on Electrocortical (ECoG) power spectrum power in rats at various times after administration. (A) short term (180 min) and (B) long term (5 days) evaluation of ECoG power changes.

In the short term evaluation set of experiments, the administration of saline did not induce any modification in ECoG total spectrum power values in rats exposed to acoustic stimulation with respect to non-stimulated animals (Fig 1A).

The systemic administration of MDMA (3 mg/kg) was not able to modify ECoG total spectrum power values with respect to saline treated animals. On the contrary, in MDMA-treated (3 mg/Kg) animals, acoustic stimulation induced a significant reduction in ECoG total spectrum power with respect to saline -sound off (P < 0.001; F = 0.92), saline -sound on (P < 0.001; F = 0.94) and MDMA-sound off (P < 0.001; F = 1.2) treated animals (Fig. 1A).

MDMA administered at the dose of 6 mg/kg -sound off induced a marked decrease of ECoG total spectrum power in comparison to control (saline-treated sound off; P < 0.001; F = 0.89) and MDMA (3 mg/Kg) -sound off (P < 0.001; F = 0.78) group. Sensorial stimulation enhanced ECoG activation with respect to the control (saline-treated -sound on; P < 0.001; F = 0.96), MDMA (3 mg/Kg) -sound off/sound on (P < 0.001; F = 0.95/P < 0.001; F = 0.82) and MDMA 6 mg/Kg -sound off (P < 0.001; F = 0.97) (Fig. 1A).

In all the experiments the effects of MDMA became evident within 1–3 min after the treatment.

### Long term evaluation

In the long term evaluation set of experiments, animals treated with saline-sound off did not show any change of ECoG total spectrum power values with respect to rats treated with saline-sound on, MDMA 3 mg/Kg -sound off, 6 mg/Kg -sound off and MDMA 3 mg/kg -sound on. These effects lasted 120–180 min after administration and the evaluation of ECoG total spectrum power 24 h, 3 and 5 days after treatment, did not evidence any difference with respect to the control (saline-treated) group (Fig. 1B).

On the contrary, the long term evaluation of ECoG parameters in animals treated with the higher dose of MDMA (6 mg/kg) -sound on, evidenced a significant decrease of total spectrum power values 24 h, 3 and 5 days after treatment with respect to the control (saline-sound off; P < 0.001; F = 0.90), saline-sound on (P < 0.001; F = 0.88), MDMA (3 mg/Kg) -sound off (P < 0.001; F = 0.87)/-sound on (P < 0.001; F = 0.95) and to MDMA (6 mg/Kg) -sound off (P < 0.001; F = 0.94) treated animals.

## Discussion

The most relevant finding in these experiments is that rats exposed to an acoustic stimulation (95 Db) that *per se *does not modify the electrocortical parameters evaluated, show, after the administration of MDMA, a marked increase in electrocortical activity with respect to animals treated with the same dose of drug but in absence of sensorial stimulation.

In particular, the lower dose of MDMA used (3 mg/Kg) was not able to modify electrocortical parameters considered only in absence of sound stimulation when, in the same treatment group, the administration of sound significantly reduced the ECoG power. In addition, the administration of a single dose of 6 mg/kg of MDMA, induced (in the presence of acoustic stimulation), significant stimulation of the electrical activity of the brain cortex lasting for five days after the administration of the drug.

The mechanisms underlying these differences in the duration of effects of similar treatments (MDMA 3 or 6 mg/kg -sound on) remain obscure; however, one might speculate that the higher (6 mg/kg) dosage of MDMA used in the present study might have produced a comparable cross-sensitisation of the animals to react with higher (and long lasting) electrocortical activity to acoustic stimuli.

Also the neurochemical basis of the synergism between noise exposure and MDMA call for more in-depth studies aimed at disclosing the fine mechanisms underlying this enhancement. In fact it has been well demonstrated that exposure to MDMA produces in mice long-lasting EEG changes and latent brain hyperexcitability, as shown by persistent changes in baseline and activated EEG, seizure facilitation and latent metabolic hyperactivity and that these effects are concomitant with monoamine depletion within limbic regions and basal ganglia [8], and studies focused on the basal ganglia circuitry [11], evidentiate that neurotoxicity affect either serotonin (5-HT) or/and dopamine (DA) nerve endings.

Indeed, the data available in literature mainly relate to tests on animals, in which the short- and long-term neurotoxic effects of MDMA are evaluated following administration of high doses (ranging from 10 to 20 mg/kg) which in some studies are repeated for as long as seven consecutive days [1,5,8,10].

It has been also well demonstrated that acoustic stimulation combined with ecstasy produces a selective enhancement of neurotoxicity (nigrostriatal damage) [8] and cardiotoxicity [12] in the mouse.

Despite the increasimg number of evidences demonstrating the synergism between noise and MDMA in inducing toxical effects, it is very difficult to indicate the mechanism underliyng these effects. The persistence of the electrocortical effects need in-depht studies aimed at elucidating the link between serotonergic and acoustic systems and the biochemical changes induced by MDMA and noise – treatment.

Our experiments evaluated the effect on animals of low doses of MDMA associated with sensorial (acoustic stimuli) comparable to those occurring in human life within young people's social gatherings of the "rave" or "techno" type, whose habitués are known to regularly take this type of drug, especially during parties chiefly characterised by strong sensorial stimulations.

## Conclusion

Taken together, our data demonstrate that MDMA, even taken in low doses, is capable of reducing the total power of the electrocorticographic spectrum, a parameter for the evaluation of the activation of the telencephalic cortex, in rats.

In our experimental conditions this activation spontaneously reverts in the absence of sensorial stimuli, whereas it persists for several days if, in addition to MDMA, the animals are exposed to acoustic stimulation.

We can therefore state that the effects of this drug could be potentiated by relatively common environmental factors and stress the potential danger for man of substances that have been so "popularly" accepted as relatively "safe" owing to their "short term" effects.

## Methods

Adult male Wistar rats weighing 250–280 g (three months old) were obtained from Charles River (Milan, Italy) and housed in a temperature (20°C) and humidity (60%)-controlled colony room. The colony, in pathogen-free conditions, was maintained in a 12 h light/dark cycle with light on at 7.00 a.m. with both laboratory food and tap water available *ad libitum*.

The experimental protocol and procedures used meet the guidelines of the Ministry of Health (G.U. n. 40, Feb. 18, 1992) for the use of laboratory animals in Italy.

Rats were anesthetized with chloral-hydrate (400 mg/kg i.p., Sigma Chemical Co., St. Louis, MO, USA) and placed in a Kopf stereotaxic apparatus. For each rat, four handmade steel epidural electrodes were inserted through a hole drilled in the skull onto each fronto-parietal cortex 2 mm behind the bregma and ± 2 mm laterally to the midline. In detail, they were produced from a 1.5-mm diameter wire, which was molded and flattened on one side, and was then bent to 90°. The flattened end of the electrode possessed a recording surface of 2.25 mm^2 ^and was placed right below the skull through a burr hole. The electrodes were kept in place by dental acrylic cement and jeweler screws, for chronic EEG recordings (see [11]). The animals were allowed 1 week to recover before testing.

Before experiments, the animals were placed individually in a sound-proof Mercury chamber modified to allow simultaneous ECoG recording (Scalone, Italy) and allowed 30 min to acclimatize to the new environment.

In awake, freely moving animals, ECoG traces were continuously recorded for 60 min before and 180 min after drug injection by connecting the electrodes to an 8 channel EEG recorder (ERA-9; OTE Biomedica, Florence, Italy). For long-term evaluation, animals were returned to testing in the same conditions 24 h, 3 and 5 days after treatment. Spontaneous and treatment-induced changes in the domain of the total ECoG spectrum power (0.25–16 Hz) were monitored continuously for periods of 30 seconds. Computerized quantitization of changes in ECoG signal amplitude (μV) was obtained with the aid of a Berg-Fourier analyser (OTE Biomedica, Florence, Italy). For statistical purposes, ECoG signal amplitude was expressed as mean ± s.e. mean percentage changes from control amplitude. The resulting means from control and test experiments were evaluated statistically for differences by prior two way ANOVA followed by Tukey Test. The parameters evaluated were: stimulation (sound on vs sound off) and treatment (saline vs dose of MDMA used). ECoG activity was monitorized 24 h, 3 and 5 days after by repeating recording in the same conditions but omitting pharmacological treatment or acoustic stimulation.

For the sensorial stimulation rats were exposed for all the duration of the first electrocortical recording (60 min before and 180 min after administration; in no case animals were exposed to another session of acoustic stimulation) to continuous white noise produced by two loudspeakers (set at 95 dB) driven by a white-noise generator (0–26 kHz), which was installed 30 cm apart from the cage. The sound level was monitored by a sound level meter (Quest electronics, 215) and it was uniform throughout tha cage. The level of loud noise was selected in order to mimic the same intensity to which humans are exposed in the discoteques (95 dB is the maximum intensity permitted from the Italian law).

3,4-Methylenedioxymethamphetamine, purchased from SALARS (Como, Italy), was dissolved in normal saline. Rats (n = 5 for each group) were randomly assigned to one of the regimens, each receiving one intraperitoneal injection of either normal saline (0.5 ml) or MDMA (3 or 6 mg/kg; 0.5 ml) with sound (95 Db) on or off.

Thus, the treatment regimens were as follows: Sound off + saline; Sound on + saline; Sound off + MDMA; Sound on + MDMA. The administration of sound was started 60 min before the injection of saline or MDMA.

## Authors' contributions

MI coinceived and coordinated the study and performed electrocortical analisys. SB, DP and MCZ carried the study and performed the statistical analysis. SG participated in design and coordination of the study and helped to draft the manuscript. FSC and DR supervised the study. All authors read and approved the final manuscript.

## Figures and Tables

**Figure 2:**
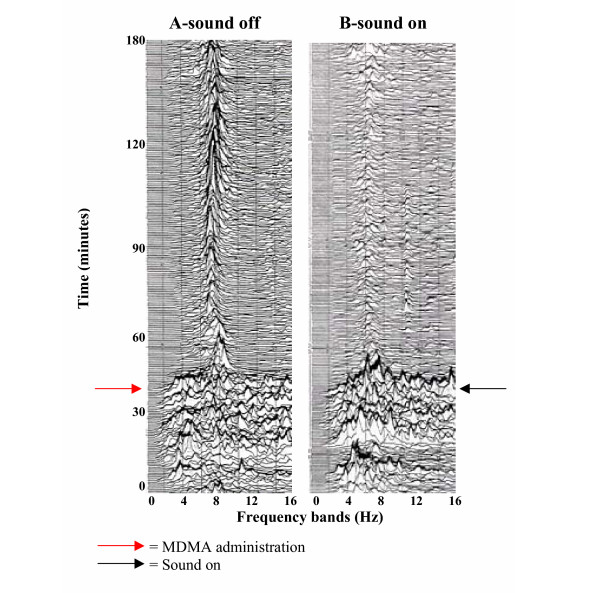
**Electrocorticographic activity changes in dependence of sensorial stimuli in MDMA – treated rats**. Sequential spectral analysis illustrating the effects of (A) MDMA (6 mg/kg; i.p.) and (B) sound (95 dB) + MDMA (6 mg/Kg; i.p.) on electrocorticographic activity in rats. The ECoG activity, evaluated at various times after treatment shows a marked decrease in total power after simultaneous administration of sound (A VS B).
